# The pan-genome of *Lactobacillus reuteri* strains originating from the pig gastrointestinal tract

**DOI:** 10.1186/s12864-015-2216-7

**Published:** 2015-12-01

**Authors:** Udo Wegmann, Donald A. MacKenzie, Jinshui Zheng, Alexander Goesmann, Stefan Roos, David Swarbreck, Jens Walter, Lisa C. Crossman, Nathalie Juge

**Affiliations:** The Gut Health and Food Safety Programme, Institute of Food Research, Norwich Research Park, Norwich, NR4 7UA UK; State Key Lab of Agricultural Microbiology, Huazhong Agricultural University, Wuhan, China; Bioinformatics and Systems Biology, Justus-Liebig-Universität, Gießen, 35392 Germany; Department of Microbiology, Swedish University of Agricultural Sciences, Uppsala, S-750 07 Sweden; The Genome Analysis Centre, Norwich Research Park, Norwich, NR4 7UH UK; Department of Agricultural, Food, and Nutritional Science, University of Alberta, Edmonton, AB T6G 2R3 Canada; Department of Biological Sciences, University of Alberta, Edmonton, AB T6G 2E1 Canada; School of Biological Sciences, University of East Anglia, Norwich, NR4 7TJ UK; SequenceAnalysis.co.uk, NRP Innovation Centre, Norwich, NR4 7UG UK

**Keywords:** *Lactobacillus reuteri*, Pig, Host-specificity, Comparative genomics, Clade-specific genes, Surface adhesins, Serine-rich repeat proteins, Auxiliary secretion system

## Abstract

**Background:**

*Lactobacillus reuteri* is a gut symbiont of a wide variety of vertebrate species that has diversified into distinct phylogenetic clades which are to a large degree host-specific. Previous work demonstrated host specificity in mice and begun to determine the mechanisms by which gut colonisation and host restriction is achieved. However, how *L. reuteri* strains colonise the gastrointestinal (GI) tract of pigs is unknown.

**Results:**

To gain insight into the ecology of *L. reuteri* in the pig gut, the genome sequence of the porcine small intestinal isolate *L. reuteri* ATCC 53608 was completed and consisted of a chromosome of 1.94 Mbp and two plasmids of 138.5 kbp and 9.09 kbp, respectively. Furthermore, we generated draft genomes of four additional *L. reuteri* strains isolated from pig faeces or lower GI tract, lp167-67, pg-3b, 20-2 and 3c6, and subjected all five genomes to a comparative genomic analysis together with the previously completed genome of strain I5007. A phylogenetic analysis based on whole genomes showed that porcine *L. reuteri* strains fall into two distinct clades, as previously suggested by multi-locus sequence analysis. These six pig *L. reuteri* genomes contained a core set of 1364 orthologous gene clusters, as determined by OrthoMCL analysis, that contributed to a pan-genome totalling 3373 gene clusters. Genome comparisons of the six pig *L. reuteri* strains with 14 *L. reuteri* strains from other host origins gave a total pan-genome of 5225 gene clusters that included a core genome of 851 gene clusters but revealed that there were no pig-specific genes *per se*. However, genes specific for and conserved among strains of the two pig phylogenetic lineages were detected, some of which encoded cell surface proteins that could contribute to the diversification of the two lineages and their observed host specificity.

**Conclusions:**

This study extends the phylogenetic analysis of *L. reuteri* strains at a genome-wide level, pointing to distinct evolutionary trajectories of porcine *L. reuteri* lineages, and providing new insights into the genomic events in *L. reuteri* that occurred during specialisation to their hosts. The occurrence of two distinct pig-derived clades may reflect differences in host genotype, environmental factors such as dietary components or to evolution from ancestral strains of human and rodent origin following contact with pig populations.

**Electronic supplementary material:**

The online version of this article (doi:10.1186/s12864-015-2216-7) contains supplementary material, which is available to authorized users.

## Background

The gastrointestinal (GI) tract of vertebrates is colonised by a complex microbial community dominated by bacteria referred to as the gut microbiota. By having a profound influence on vertebrate physiology, metabolism, and immune functions, the gut microbiota plays important roles in the health of the host [1, 2]. These associations open avenues for the development of therapies that aim to restore the ecosystem, but their implementation requires a mechanistic understanding about the ecological principles that shape and regulate microbial communities [3, 4]. In contrast to microbial symbiosis in invertebrates, little is known about the basic principles that underlie symbiotic interactions in vertebrates and how they evolve [5].

*Lactobacillus reuteri*, a Gram-positive bacterial species that colonises the gut of a variety of vertebrate species, has been used as a model to determine the ecology and evolution of vertebrate gut symbionts [5]. The ecological strategies of *L. reuteri* are fundamentally different in humans and animals [6]. In rodents, pigs, chickens and horses, lactobacilli form large populations in proximal regions of the GI tract, and they adhere directly to the stratified squamous epithelium present at these sites [7–9]. In mice and rats, adherence occurs in the forestomach [10, 11], and this process appears to be important with regards to the ecological fitness of the bacteria [5]. The epithelial associations formed can be considered biofilms as the bacteria are arranged in multiple layers and are encased in a polysaccharide matrix [9, 12, 13]. In contrast, stratified squamous epithelia are absent in the human gut, and epithelial cell layers rich in lactobacilli equivalent to those found in the above-mentioned animals have not been described [6]. Rather, a more transient colonisation of the human GI tract by *L. reuteri* is likely to be mediated by mucus-binding adhesins (as discussed below), resulting in a relatively low prevalence in the human population even although this species is still considered to be autochthonous in humans [5, 6].

Using a combination of population genetics and comparative genomics, we have recently demonstrated that *L. reuteri* is composed of host-specific clades with lineage-specific genomic differences that reflect the niche characteristics in the GI tract of the respective hosts. Host adaptation of this species is supported by genetic clustering of strains originating from common or related hosts. Amplified-fragment length polymorphism (AFLP) and multi-locus sequence analysis (MLSA) with more than 100 *L. reuteri* strains isolated from humans, pigs, rats, mice, chickens and turkeys revealed that considerable genetic heterogeneity exists within the *L. reuteri* population, with distinct phylogenetic clades that reflect host origin of the strains [14]. Experiments in *Lactobacillus*-free mice to measure the ecological fitness of strains originating from different hosts supported host adaptation, as only rodent strains colonised mice efficiently [15]. Furthermore the ability of *L. reuteri* to form epithelial biofilms in the mouse forestomach of mono-associated mice was strictly dependent on the strain’s host origin [12].

Genome comparisons of *L. reuteri* strains originating from different hosts identified lineage-specific genomic content that reflects the niche differences in the GI tract of rodents and humans. The ecological significance of a subset of rodent-specific *L. reuteri* 100-23 genes was demonstrated in the context of the murine gut [12]. This mutational analysis revealed that genes encoding proteins involved in epithelial adherence, specialised protein transport, cell aggregation, environmental sensing and cell lysis contributed to biofilm formation and colonisation. In particular, the inactivation of a serine-rich repeat protein (SRRP) surface adhesin with a devoted transport system (the SecA2-SecY2 pathway) completely abrogated colonisation of the mouse forestomach, indicating that initial adhesion represented the most significant step in biofilm formation, likely conferring host specificity [12]. Similarly, host-strain specific adhesins have been recently reported in other *L. reuteri* strains as playing a key role in the interaction of the bacteria with their host. These include the mucus-binding proteins, CmbA from *L. reuteri* human strains [16, 17] and MUB from *L. reuteri* ATCC 53608, a strain isolated from pig [18–21].

Comparative genomics of *L. reuteri* strains also revealed distinct levels of genetic heterogeneity in different phylogenetic lineages. While human *L. reuteri* strains from the lineage II, F275 (JCM1112^T^/DSM20015^T^), ATCC PTA-6475 (MM4-1a), and ATCC PTA-4659 (MM2-3) possess a closed pan-genome with >99.5 % average nucleotide identity (ANI), strains from rodents possessed a larger pan-genome with a variable gene content and an ANI of around 96–97 %, and the majority of the rodent-specific genes detected by comparative genomic hybridisation (CGH) were not conserved among rodent strains [15].

The work described above has established *L. reuteri* as an excellent model to elucidate mechanisms for host-microbial symbiosis in vertebrates. However, the mechanisms by which *L. reuteri* strains specifically colonise the porcine GI tract and the evolutionary processes that resulted in host-specific phylogenetic clusters have not been elucidated. Such knowledge is relevant as *L. reuteri* is one of the most dominant species in the porcine GI tract [22] and pig-derived strains of *L. reuteri* are used as probiotics to improve pig health and well-being [23–25]. Previous MLSA indicated that porcine isolates fall within two distinct phylogenetic groups, clades IV and V, that are to a large degree host-specific, suggesting adaptation and specialisation towards the porcine host [14]. Here we performed a pan-genome analysis of six *L. reuteri* strains isolated from pigs (four from clade IV and two from clade V) with the goal to gain insight into the molecular basis for colonization of the porcine gut, identify genes marking host-specific adaptations, and determine differences between the two *L. reuteri* lineages present in pigs. Further comparison with the genomes from human and rodent *L. reuteri* strains was carried out providing novel insights into the genomic events in *L. reuteri* that occurred during specialisation to the porcine host.

## Results and discussion

### Complete genome sequence of the pig *L. reuteri* strain ATCC 53608

The *L. reuteri* ATCC 53608 genome was sequenced by 454 pyrosequencing and previously published as a draft assembly [26]. According to previous phylogenetic analyses, the ATCC 53608 strain falls into the pig-specific MLST cluster IV [14] (Table 1). Here the genome was completed and fully assembled as described in Methods to create a model reference backbone for genome comparisons of *L. reuteri* strains from pig.

**Table 1:** *L. reuteri* strains used in this study for genome sequencing and for comparative genomics

Strain (alternative name)	Host	MLST^a^ group	Country of origin	Source
ATCC 53608	Pig	IV	Sweden	Stefan Roos
lp167-67	Pig	IV	Sweden	Stefan Roos
pg-3b	Pig	IV	USA	Jens Walter
I5007	Pig	IV^b^	China	GenBank: CP006011-CP006017
20-2	Pig	V	Germany	Wolfgang Souffrant
3c6	Pig	V	New Zealand	Gerald Tannock
lpuph1	Mouse	I	USA	JGI: 2506381017
TD1	Rat	I^b^	USA	GenBank: CP006603
100-23	Rat	III	New Zealand	JGI: 2500069000
mlc3	Mouse	III	USA	JGI: 2506381016
TMW1.112	Sourdough	III^b^	Germany	JGI: 2534682347
TMW1.656	Sourdough	III^b^	Germany	JGI: 2534682350
LTH2584	Sourdough	III^b^	Germany	JGI: 2534682349
LTH5448	Sourdough	I^b^	Germany	JGI: 2571042361
JCM1112^T^ (DSM20016^T^/F275)	Human	II	Germany	NCBI: NC_010609
ATCC PTA-6475 (MM4-1a)	Human	II	Finland	JGI: 2502171170
ATCC PTA-4659 (MM2-3)	Human	II^b^	Finland	JGI: 2502171171
ATCC 55730 (SD2112)	Human	VI	Peru	NCBI: NC_015697-NC_015701
CF48-3a1	Human	VI^b^	Finland	JGI: 2502171173

^a^Multi-Locus Sequence Type analysis [14]

^b^MLST determined from genome sequence

#### Genome organisation

**Fig. 1:**
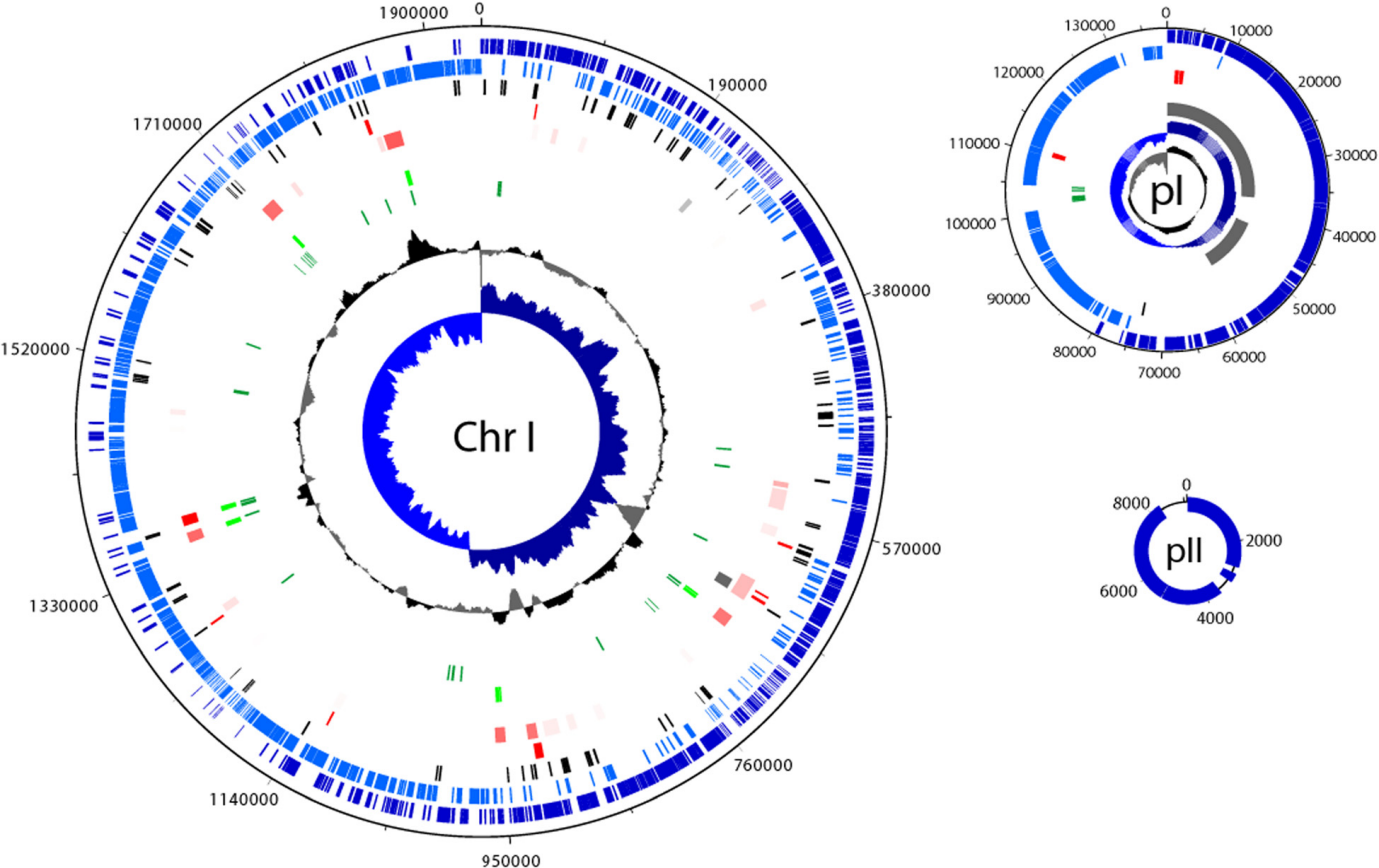
Multireplicon genome of *L. reuteri* strain ATCC 53608. Circles from outside to the centre: genes on forward strand (dark blue), genes on reverse strand (light blue), pseudogenes (black), alien genes (COLOMBO; red), alien genes (Alien_hunter; pink), PHAST bacteriophage remnants (grey), rRNA genes (light green), tRNA genes (dark green), GC content (window size 20000; step size 200; black/grey), GC skew (window size 20000; step size 200; dark blue/light blue)

The genome of *L. reuteri* ATCC 53608 consists of three circular molecules (Fig. 1 and Table 2), a chromosome of 1943635 bp and two plasmids of 138515 bp (pI) and 9093 bp (pII), respectively. The two plasmids were confirmed to be closed circular molecules by PCR with primer pairs B40/B41 (pI) and sc10-for/sc10-rev (pII) that flanked each single gap. The chromosome has an average GC content of 38.96 % compared with 38.17 % for the small and 35.84 % for the large plasmid. The Oriloc software [27] in combination with the R software package [28] was used to identify the putative *oriC* and *ter* regions based on the cumulated 3^rd^ codon position skews. In the case of the chromosome, the positive peak for the combined skew is located immediately upstream of the *dnaA* gene and we predict the origin of replication to be located in the AT rich (69.9 % A + T) intergenic region between the *rpmH* (LRATCC53608_1967) and *dnaA* (LRATCC53608_0001) genes. Allowing for one mismatch, we detected 10 DnaA boxes (TTRTCCACA) in this stretch of the genome, which were equally distributed between the forward and the reverse strands and we therefore designated the first base pair of *dnaA* as position 1 of the chromosome. The negative peak of the combined skew, indicating the putative terminus region, is located in LRATCC53608_1036 (position 1008794–1010011). A total of 1880 CDSs were identified on the chromosome, 75.6 % of which were encoded on the leading strand of chromosome replication. This strong leading strand coding preference is a typical value observed in many other Gram-positive organisms. Plasmid pI is a megaplasmid which carries a RepA/ParA type replicative system and—like the chromosome—displays strong 3^rd^ codon position skews. This plasmid carries a total of 160 CDSs, 98.8 % of which are encoded on the leading strand of replication. Besides an incomplete prophage, the plasmid also carries genes encoding enzymes for purine and pyrimidine metabolism (deoxyguanosine kinase, nucleoside deoxyribosyltransferase, thymidine kinase), a nicotinamide mononucleotide transporter, a *dnaE* and tRNA genes (tRNA-Thr, -Met, -Gly, -Asn, -Trp and -Arg). Though none of these can explain the stable maintenance of this megaplasmid, resulting in a considerable burden on the cell’s metabolism, as they are all additional copies of genes that are also present on the chromosome. A toxin–antitoxin system encoded on this plasmid (LRATCC53608_pI137-_pI138) is the most likely explanation for its stability. Plasmid pI also encodes a large number of hypothetical proteins. Plasmid pII carries six CDSs and is potentially mobilisable by means of a predicted relaxase/mobilase. A putative replication protein shares low sequence similarity with replication proteins from other lactobacilli. Interestingly, the completed genome sequence confirmed plasmid isolation data that strain ATCC 53608 lacks the 10251 bp plasmid pLUL631 [EMBL:HF570055] which is present in parental strain 1063 [29] (Additional file 1: Figure S1A) and which encodes resistance to both erythromycin (*ermB*) and streptogramin A (*satG*).

**Table 2:** Genome overview of *L. reuteri* ATCC 53608

DNA molecule	Chromosome	Plasmid pI	Plasmid pII
Number of bases	1943635	138515	9093
%GC content	38.96	35.84	38.17
Number of CDS	1880	160	6
Gene Density	0.895	1.147	0.659
Average gene length	934	734	1278
Coding percentage	83.7	84.2	84.3
Pseudo classifier	139	1	0

#### Predicted primary metabolism

Based on the genome annotation, *L. reuteri* ATCC 53608 has very limited amino acid synthesis capabilities. This strain should be able to synthesise serine and glycine *de novo* from pyruvate using serine dehydratase (EC 4.3.1.17), which catalyses pyruvate-serine interconversion. The α and β chains of this protein are encoded by *sdaAA* and *sdaAB* (LRATCC53608_0899 and _0898), respectively. Serine could subsequently be converted to glycine by serine hydroxymethyltransferase encoded by *glyA. L. reuteri* cannot carry out *de novo* synthesis of the aspartate family of amino acids as it does not carry a gene for pyruvate carboxylase, however it should be able to synthesise lysine and asparagine from aspartate. The strain also lacks the gene for glutamate synthase but can interconvert glutamate and glutamine and carries a *proBAC* operon enabling the synthesis of proline from glutamate. Most pathways for vitamin biosynthesis are incomplete (biotin, cobalamine, pantothenate, pyridoxal phosphate, nicotinate and thiamine). Complete pathways were only found for the biosynthesis of folate and possibly riboflavin. *L. reuteri* ATCC 53608 cannot synthesise lipoic acid, but appears able to produce coenzyme A from pantothenate and NAD^+^ and NADP^+^ from nicotinamide mononucleotide, for whose uptake the genome encodes two transport proteins (LRATCC53608_0249 and LRATCC53608_pI116). Purine and pyrimidine biosynthetic pathways are present in *L. reuteri* ATCC 53608 and the formation of deoxyribonucleotides is not hampered by oxygen, as the strain possesses an oxygen-requiring class Ib enzyme encoded by *nrdE2* and *nrdF1* (LRATCC53608_1673 and _1674, respectively) and the necessary auxiliary proteins NrdH and NrdI (LRATCC53608_1672 and LRATCC53608_0276), in addition to oxygen-sensitive class III ribonucleotide reductases encoded by *nrdD* and *nrdG* (LRATCC53608_0711 and _0712, respectively). The genes encoding these different ribonucleotide reductases appear organised in two operons (*nrdDG* and *nrd*HE2F1), the first gene of which is preceded by one or two NrdR boxes, respectively, that according to the RegPrecise database [30] are typical for *Lactobacillaceae*, indicating that these genes are regulated by the negative transcriptional regulator NrdR (LRATCC53608_0784).

#### Horizontal gene transfer (HGT): pseudogenes, alien genes, IS elements and bacteriophages

The genome of *L. reuteri* ATCC 53608 contains 140 pseudogenes in total, representing around 6.8 % of the total gene number, thus significantly higher than the 1–5 % average figure reported for other bacterial genomes [31]. The Colombo SIGI-HMM software [32] predicts 1.5 % of the total gene content of strain ATCC 53608 to originate from HGT (Fig. 1). The genome of ATCC 53608 was found to be moderately repetitive, containing 15 different insertion sequence (IS) elements from seven IS element families, particularly of the IS30 family (Additional file 2) contributing a total of 126 genes (including 37 pseudogenes) or 6.2 % of all genes encoded in the genome of *L. reuteri* ATCC 53608. Using the IS element sequences we tried to identify the insertion sites and in many cases were able to create target site sequence logos using WebLogo 3 [33] on the basis of the direct repeats flanking the IS elements (Additional file 1: Figure S2). The PHAST software [34] identified three extended regions of bacteriophage origin in the genome of *L. reuteri* ATCC 53608. The first is located on the chromosome (position 650477–661412*)* and the latter two are located in close vicinity to each other on plasmid I (positions 1–36485 and 43193–57464) (Fig. 1).

### Assembly and chromosomal features of four additional pig strains of *L. reuteri*

**Fig. 2:**
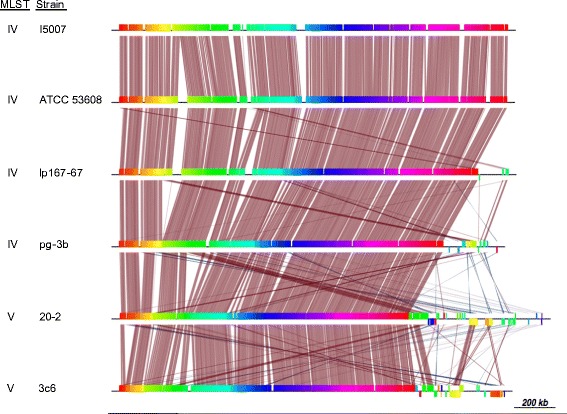
Genome conservation in the six pig strains of *L. reuteri*. From top to bottom, the Mauve-alignment backbone of the draft genome sequences is shown as follows: I5007, ATCC 53608, lp167-67, pg-3b, 20-2 and 3c6. Blocks of sequence conservation are denoted by burgundy lines, whilst blocks as calculated by Mauve are shown in alternating colours

We selected four additional *L. reuteri* strains isolated from pigs for genome sequencing, choosing strains from both pig-specific MLST clades IV (strains lp167-67 and pg-3b) and V (strains 20-2 and 3c6) [14] (Table 1, which includes their different geographical locations). Based on MLST analysis of seven housekeeping genes, type V strains are located phylogenetically close to, although distinct from, *L. reuteri* strains of human/avian origin type VI, whereas those from type IV fall between human and rodent isolates of types II and III, respectively [14]. High coverage Illumina data were obtained for these four *L. reuteri* strains and draft assemblies were generated as described in Methods. Here we assigned taxon data to each separate contig and plotted % GC against the % coverage. During the assembly process, a very low proportion of the reads was assigned to a few contaminating contigs of eukaryotic origin; additionally, in one of the assemblies (namely 20–2), it was possible to discern a related *Lactobacillus* sequence at a very low coverage, which gave rise to many short contigs that were removed from the final assembly. The length of the final assembly and representation of expected genes is presented in Table 3. Each of the assemblies possessed ≤201 contigs of >100 bp at high coverage with a median contig length of 7676, 5185, 6647 and 5386 bp for pg-3b, 3c6, 20-2 and lp167-67, respectively. The chromosomes of the completed and draft assembly strains showed syntenic conservation as aligned by Mauve [35] and by MUMmer v3.0 [36] with relatively few regions of difference observed (Fig. 2). Each draft genome was compared to the ATCC 53608 genome by measuring the % ANI—lp167-67 (99.6 %), pg-3b (99.2 %), 3c6 (95.2 %) and 20-2 (95.1 %). All their ANI values were >95 %, confirming their identities as members of the same species [37], *viz. L. reuteri*. The slightly lower ANI values of just over 95 % for strains 3c6 and 20-2 reflected their presence in a separate phylogenetic clade to the others.

**Table 3:** Draft genome assembly statistics for four *L. reuteri* strains isolated from pig GI tract

Strain	No. of contigs >100 bp	Shortest contig (bp)	Longest contig (bp)	Mean contig length (bp)	Trimmed mean contig length (bp)	Median contig length (bp)	Total genome size (Mbp)	Minimum number of CDS
lp167-67	134	203	144847	14970	14104	5386	2.021	2019
pg-3b	104	213	123900	18056	17202	7676	1.896	1847
20-2	188	243	95312	11843	11458	6647	2.238	2272
3c6	201	204	67417	9604	9362	5185	1.935	1918

### The pig *L. reuteri* pan-genome

The complete ATCC 53608 genome was used as a model reference backbone to compare the assemblies of the additional four draft genomes of *L. reuteri* from pigs (lp167-67, pg-3b, 20-2 and 3c6). In addition, the recently completed genome of another pig *L. reuteri* strain, I5007 (isolated from the colonic mucosa of a weaning piglet in China) [38], was included in genome comparisons. The combination of these six strains was referred to as the pig *L. reuteri* pan-genome.

#### Analysis of the pig chromosomal core and accessory genomes

**Fig. 3:**
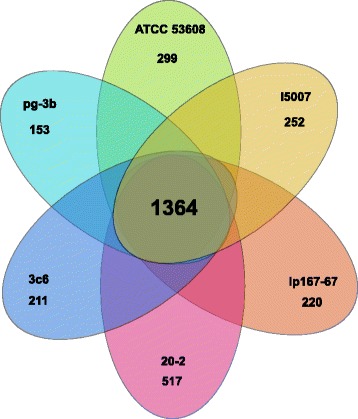
Pan-genome of the six pig *L. reuteri* strains. Each genome is represented by a coloured oval. The number in the centre represents the core gene set shared among the six pig strain genomes expressed as orthologous gene clusters per genome. The numbers of genes unique to each genome are indicated by the outer values. In addition, 799 orthologous gene clusters were shared by at least two genomes, contributing to a current pig strain pan-genome of 3373 orthologous gene clusters. Short genes (<150 bp) were excluded from the analysis

**Fig. 4:**
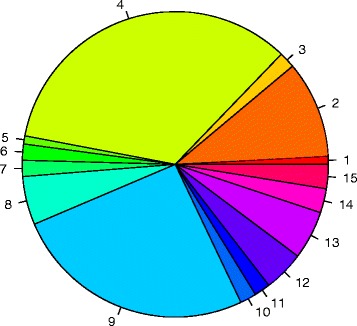
Predicted functions encoded by genes of the accessory genome from six *L. reuteri* porcine isolates. Functions listed are: 1, Cofactors; 2, Cell wall and capsule; 3, Virulence and defence; 4, Phages and mobile elements; 5, RNA metabolism; 6, Nucleosides and nucleotides; 7, Protein metabolism; 8, Regulation; 9, DNA metabolism; 10, Fatty acids and lipids; 11, Respiration; 12, Stress response; 13, Amino acids and derivatives; 14, Sulphur metabolism; and 15, Carbohydrates

The backbone genetic information is defined as the genetic information common to all strains tested, which is also referred to as the ‘core genome’ and in this case represents genes present in all of the above *L. reuteri* strains isolated from pig. The core set from these six genomes contained 1364 orthologous gene clusters as determined by OrthoMCL analysis [39] and 1210 gene clusters, comprising 1652 “unique” genes that occurred at least once in individual genomes—the difference between the latter two values being due to the presence of at least two gene copies in some genomes. Another 799 gene clusters were shared by at least two genomes, giving a total of 3373 orthologous gene clusters in the current pig strain pan-genome (Fig. 3 and Additional file 1: Figure S3A). Very short predicted genes (<150 bp) were excluded from the analysis. Genes variable among pig strains make up the accessory genome. These regions were parsed from the pig isolates at the DNA level using the Panseq program [40]. A successive round of bioinformatic purification of DNA sequence from the output of the Panseq pipeline was carried out using MUMmer [36] to further refine the data. The accessory regions were annotated separately and viewed functionally as a pie chart (Fig. 4). Compared to the functions of the whole genomes, these regions are rich in genes encoding cell wall-associated proteins, capsule/exopolysaccharide (EPS) biosynthetic enzymes, phage-related functions, mobile elements and DNA metabolic enzymes, whilst being low in essential metabolism genes. This is similar to the situation with rodent strains of *L. reuteri* where many cell surface proteins, proteins involved in surface polysaccharide biosynthesis and prophages fall within the accessory genome [15].

The six genome sequences from porcine origin were examined further for the presence of clustered regularly interspaced short palindromic repeats (CRISPRs) against the CRISPRdb database [41] using the CRISPRFinder tool [42] and any such sequences compared using the CRISPRcompar website [43]. However, although questionable CRISPRs were identified for all genomes, no confirmed CRISPRs were uncovered by this analysis. Questionable CRISPRs include small CRISPRs of two to three direct repeats and structures where the repeats are not 100 % identical. In the ATCC 53608 finished genome, questionable CRISPRs were located at 893116–893231 bp and comprised a set of two virtually identical direct repeats. An almost identical situation occurs at 868686–868801 bp in strain I5007. However, no CRISPR-associated (*cas*) gene hits were uncovered upstream or downstream of that region in either ATCC 53608 or I5007, which is located directly downstream from the accessory secretion system, a genetic region likely acquired by HGT.

#### *Analysis of plasmids in porcine* L. reuteri *strains*

The putative plasmid DNA sequences of the four pig draft genomes were aligned to plasmid sequences of pI and pII from *L. reuteri* ATCC 53608, the sequence of plasmid pLUL631 from parental strain 1063 and the six plasmid sequences from strain I5007 and compared. This method was used to extract potential plasmid sequences as contigs from the total assembly of the four strains. Without exception, all of the contigs extracted from each of the strains were present within the respective accessory regions that had been identified previously in comparison to strain ATCC 53608. Efforts to compare the gene annotations from the *L. reuteri* ATCC 53608 plasmids to those of the newly identified sequences were unsuccessful, in line with the lack of DNA sequence alignment, and supporting the findings that the plasmid DNA is heterogeneous and relatively unrelated in each of these strains. Further comparisons of the plasmids of ATCC 53608 with those from the other *L. reuteri* pig strains did not indicate large conservation amongst the plasmids of this species. Rather the plasmids appear isolate-dependent, which correlates with the results of plasmid isolation from these strains (Additional file 1: Figure S1B).

#### *Comparative genomics of* L. reuteri *genomes from different host origin*

In order to gain insights into the subset of genes involved in adaptation to the porcine gut, we performed a comparative genomic analysis of 20 *L. reuteri* strains that are currently available, which originate from the pig (p), human (h) and rodent (r) GI tracts and from sourdough (s). These strains were lp167-67(p), pg-3b(p), 3c6(p), 20-2(p), ATCC 53608(p), I5007(p), JCM1112^T^(h), DSM20016^T^(h), ATCC PTA-6475 [MM4-1A] (h), ATCC PTA-4695 [MM2-3] (h), CF48-3A1(h), ATCC 55730 [SD2112] (h), TD1(r), lpuph1(r), mlc3(r), 100-23(r), TMW1.112(s), TMW1.656(s), LTH2584(s) and LTH5448(s). A set of 19 of the available 20 genomes was used in the analysis, whereby JCM1112^T^ and DSM20016^T^ were considered as a single genome (that of JCM1112^T^) since these two strains are derived from the same isolate (F275). The core set of these 19 genomes contained 851 orthologous gene clusters by OrthoMCL [39] analysis, while there were 1890 gene clusters, comprising 3479 “unique” genes that occurred at least once in individual genomes – the difference between the latter two values again being due to the presence of at least two gene copies in some genomes. Another 2484 gene clusters were shared by at least two genomes, giving a total *L. reuteri* pan-genome of 5225 orthologous gene clusters (Additional file 1: Figures S3B and S4). A circular comparison of the genomes of the six strains isolated from pig with genomes from a selection of seven other *L. reuteri* strains from human and rodent is shown in Additional file 1: Figure S5.

Homologous genes were calculated using *L. reuteri* ATCC 53608 as the reference strain by reciprocal best match analysis requiring a 40 % match by FASTA searches over 80 % of the target protein length. This required the target protein to be predicted almost in entirety using current gene prediction software Prodigal [44] and Glimmer3 [45]. With this method, unpredicted proteins, either due to unusual base composition or to fragmentation at the contig level, would lead to a negative result. Using protein sequences derived from the genomic data, three additional analyses (see Methods) using the Joint Genome Institute’s Integrated Microbial Genomes (IMG/ER) Phylogenetic Profiler tool [46], reciprocal FASTA with the annotation software Artemis [47, 48], and OrthoMCL [39] all indicated that there were no genes conserved in all six pig-derived genomes that were absent in the genomes of *L. reuteri* strains from other hosts. Therefore our analyses showed that there are no pig-specific genes.

**Fig. 5:**
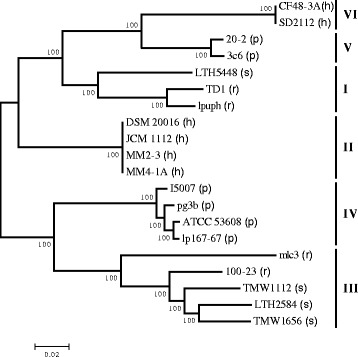
A maximum likelihood phylogenetic tree of the core genome alignment of 20 *L. reuteri* genomes. Concatenated core gene sequences were compared. The tree was bootstrapped with 1000 replicates, shown as percentages at each branch. Numbers I-VI represent the original MLSA-based groupings. The host origin of each strain is denoted in parenthesis after the strain name as follows: (h) human, (p) pig, (r) rodent and (s) sourdough

Previous MLSA revealed that pig isolates fall into two distinct clades [14]. A phylogeny based on the core genes of 20 strains of *L. reuteri* (Fig. 5) confirmed the phylogenetic clusters and their membership. Clade IV contains strains I5007, ATCC 53608, lp167-67 and pg-3b, and is more closely related to the rodent cluster III, while clade V, which is more closely related to human/poultry strains of clade VI, contains strains 20-2 and 3c6. As reported previously [14], the geographical origin of the strains was not related to the phylogenetic clades since *L. reuteri* strain I5007 was isolated in China [38] but found as a close relative of ATCC 53608 (parental strain designated 1063), isolated in Sweden [49]. These findings indicate that the co-evolution of *L. reuteri* with pigs has resulted in the formation of two vastly distinct phylogenetic clusters, which suggests that evolution of these two populations, although driven by the porcine host (thus the confinement of the clusters to pig isolates), was subjected to different selective pressures that led to their separation. The distinct evolutionary trajectories could also have resulted in the absence of common genes that reflect host adaptation among porcine strains. The relatedness of pig clade IV with rodent clade III strains, and of pig clade V with human/poultry clade VI strains may reflect their evolution from ancestral strains of rodent and human origin, respectively, following contact with pig populations.

To gain insight into this diversification process, we identified genes that were specific to the two individual clades, and conserved among the strains. This analysis revealed 10 lineage IV-specific genes encoding proteins >60 amino acids. Among these genes was one (LRATCC53608_0212) encoding a surface protein of 1166 amino acids in length that contained five mucus-binding (MucBP) domains (Table 4). In addition, cluster IV strains contained three linked genes (LRATCC53608_0113-_0115) encoding a TetR family transcriptional regulator, a conserved hypothetical protein with a DHS-like NAD/FAD-binding domain and an NAD-dependent protein deacetylase of the SIR2 family whose functions are as yet unknown. The two clade IV-specific esterase-lipases (LRATCC53608_0604 and _0605) could be involved in the utilization of lipids in the pig’s diet but on closer examination appeared to be two parts of the same pseudogene. An interesting feature of the clade-IV-specific genes was that they had very high levels of homology (>99 % aa identity) among the four different strains, which supports that they are highly conserved among porcine isolates of this clade.

**Table 4:** Genes specific to *L. reuteri* pig clades IV and V

Locus tag	Gene product or putative function	Encoded protein (aa)	COG	pfam
A. Clade IV-specific genes (strain ATCC 53608 nomenclature)				
LRATCC53608_0113	TetR family transcriptional regulator	197	-	00440
LRATCC53608_0114	Conserved hypothetical protein with DHS-like NAD/FAD-binding domain	226	-	-
LRATCC53608_0115	NAD-dependent protein deacetylase; SIR2 family	324	0846	-
LRATCC53608_0124	NAD-dependent epimerase/dehydratase; NmrA-like family	208	2910	13460
LRATCC53608_0212	Secreted LPXTG cell wall anchor protein with X5 MucBP domains; putative mucus-binding adhesin	1166	-	06458 00746
LRATCC53608_0604	Lysophospholipase; esterase-lipase superfamily; possible pseudogene with LRATCC53608_0605	197	2267	12697
LRATCC53608_0605	Lysophospholipase; esterase-lipase superfamily; possible pseudogene with LRATCC53608_0604	82	-	12146
LRATCC53608_0617	B3/4 domain-containing protein; tRNA synthetase (Phe); tRNA ligase	237	3382	03483
LRATCC53608_1334	Mid-1-related chloride channel protein with LPXTG cell wall anchor; possible truncated pseudogene lacking a secretion signal	122	-	00746
LRATCC53608_1865	Phage integrase; possible truncated pseudogene	189	-	00589
B. Clade V-specific genes (strain 20-2 nomenclature)				
LR202_00053	Helix-turn-helix protein; putative transcriptional regulator	64	-	12728
LR202_00355	Blue copper oxidase CueO precursor; cell cycle control, ion transport, cell surface biogenesis	512	2132	07731 07732
LR202_00455	Poly(glycerol-phosphate) α-glucosyltransferase (EC 2.4.1.52); cell wall biosynthesis; DUF1975	499	0438	00534 09318
LR202_00456	Poly(glycerol-phosphate) α-glucosyltransferase (EC 2.4.1.52); teichoic acid synthesis; DUF1975	518	-	09318 00534
LR202_00788	Hypothetical protein	142	-	-
LR202_00789	NADPH:quinone reductase/Zn-dependent oxidoreductase	325	0604	08240 00107
LR202_00791	Acetylornithine deacetylase (EC 3.5.1.16)/lysine biosynthesis; zinc peptidase M20	380	0624	01546 07687
LR202_00792	Mobile element protein; transposase	423	3464	14690 01610
LR202_00802	Reticulocyte binding protein; type II restriction modification DNA methylase *Eco*57I	312	-	07669
LR202_00803	TaqI-like C-terminal specificity domain; type II restriction ^m6^A DNA methyltransferase	174	-	12950
LR202_00923	TetR family transcriptional regulator	227	-	00440 14278
LR202_00927	Hypothetical protein	172	-	-
LR202_00992	Pyruvate carboxyl transferase (EC 6.4.1.1); ATP-binding domain	210	-	02786 00289
LR202_01101	Phage integrase/recombinase	379	4974	00589
LR202_01102	Phage-associated #P4-type DNA primase	478	-	-
LR202_01104	Rlx-like relaxase/mobilisation protein	485	-	03432
LR202_01105	Hypothetical protein	112	-	-
LR202_01106	Hypothetical protein	250	-	-
LR202_01429	Secreted protein with Ser/Ala-rich surface protein repeats, SEC10/PgrA surface exclusion domain and LPXTG cell wall anchor; Rib/alpha-like repeat	1133	-	00746 08428
LR202_01598	Secreted protein with SEC10/PgrA surface exclusion domain; possible pseudogene lacking an LPXTG cell wall anchor	541	-	-
LR202_01610	Conserved hypothetical integral membrane protein; possible Mg-transporting ATPase (P-type) or dolichyl-phosphate-mannose-protein mannosyltransferase PMT-2 superfamily	499	0474	-
LR202_02163	Secreted protein with Ser/Ala-rich surface protein repeats, SEC10/PgrA surface exclusion domain and LPXTG cell wall anchor; Rib/alpha-like repeat	1084	-	00746 08428

Of the 22 genes encoding proteins >60 amino acids that were specific to pig cluster V (Table 4), several represented mobile genetic elements, including transposons and phage-related sequences. However, four lineage-specific surface proteins were detected, three of which contained a SEC10/PgrA surface exclusion domain, found in Gram-positive adhesins such as SpyAD from Group A Streptococci and implicated in cell adhesion [50]. Two of these SEC10/PgrA proteins (homologues of LR202_01429 and _02163) also contained a Rib/alpha-like repeat from the Rib adhesin of Group B Streptococci [51]. Two genes encoding glycosyltransferases (predicted to be involved in biosynthesis of surface carbohydrates or protein glycosylation) were also identified. In addition, a putative copper oxidase and a magnesium-transporting ATPase were detected, suggesting interactions of this *L. reuteri* lineage with metal ions. Several hypothetical proteins were found with high homology among the two strains. Interestingly, one of these proteins (LR202_00788) showed 97 % aa identity to a protein from *Lactobacillus amylovorus*, which is indicative of HGT from another dominant member of the pig microbiota [22], suggesting an important function in the pig gut.

Previous pan-genome analysis of *L. reuteri* rodent and human strains revealed that the *pdu*-*cbi*-*cob*-*hem* cluster was conserved within human strains, and the cluster was absent in most rodent strains [5, 15]. This cluster codes for cobalamin (vitamin B12) biosynthesis, glycerol utilization, propanediol fermentation and production of the antimicrobial compound reuterin [5, 52]. The genetic region encompassing the reuterin and vitamin B12 biosynthetic pathways is located at 293889–338218 bp on porcine reference isolate *L. reuteri* ATCC 53608. As in rodent strains, it appears that this *pdu-cbi-cob-hem* genomic island is present only in a subset of porcine strains, confirming previous findings [5, 15]. Out of the four strains from clade IV, only ATCC 53608 and lp167-67 possess this genomic island, while it is present in both clade V strains 20-2 and 3c6 studied ─ although this island is not conserved in other members of pig clade V [5, 15]. Thus it appears that the possession of the reuterin genomic island does not correlate directly with the relationship of the genetic backbone. As with the rat isolate 100-23, there is evidence that the cluster has been deleted from the genome of pig isolate I5007 through the action of mobile elements.

### Molecular determinants of host recognition

In order to gain further insight into the molecular determinants of host-strain specificity, we focused on the genomic analysis of the major cell surface components that have been implicated in the interaction of *L. reuteri* strains with the host *in vivo*.

#### Cell surface proteins (including adhesins)

Several surface proteins of *L. reuteri* that are involved in colonisation by binding to epithelia, epithelial cells, or mucus have been functionally characterised [12, 15, 19–21, 53]. Proteins such as MUB and the large surface protein (Lsp) contain LPXTG cell wall anchoring motifs, are extremely large, contain multiple repeated motifs and resemble adhesins of pathogenic microbes [16, 18, 54].

Here, a total of 41 surface protein-encoding genes and three conserved pseudogenes were predicted from the completed genome of strain ATCC 53608 (Additional file 3). Potential homologues were identified in the other pig strains from the annotation and sequence read data from the draft assemblies by alignment to ATCC 53608 using Bowtie 2 [55] and checked separately by Burrows-Wheeler Aligner software, BWA [56]. Using BEDTools [57], the percentage of each protein coding sequence covered was determined in the four pig strain draft genomes and results are displayed in a heatmap (Additional file 1: Figure S6) with the characteristics of the proteins. Many of these surface proteins are predicted to be involved in epithelial adhesion and biofilm formation. MUB was found only in strain ATCC 53608 but a few other MucBP (Pfam PF06458) domain-containing proteins were identified, some as conserved pseudogenes, and two were found to be pig clade IV-specific (homologues of LRATCC53608_0212 and LRATCC53608_0767-_0769). Other putative surface proteins found only in ATCC 53608 were LRATCC53608_0656, _0662 and _0644 although the significance of these is unknown. Several proteins with one or more lysin motif (LysM/CBM50), a domain involved in peptidoglycan binding [58], were present in all five pig strains analysed, including homologues of LRATCC53608_1570, which are identical to Lr_71416 from rodent strain 100-23. LysM-containing proteins Lr_71416 and Lr_70152 are putative aggregation promoting factor proteins that have been implicated in biofilm formation and colonisation of strain 100-23 in mice [12], suggesting a similar role in the pig host. In the heatmap of Additional file 1: Figure S6, there is therefore a clear difference between the two representatives of clade IV, pg-3b and lp167-67, and the two representatives of clade V, 20-2 and 3c6, with respect to the presence or absence of certain surface proteins. On the other hand, the other member of clade IV that was used as the reference strain, ATCC 53608, possessed additional surface proteins not found in strains pg-3b and lp167-67.

One interesting putative surface protein, not included in the above analysis because of its lack of a consensus secretion signal, was the DUF1542 repeat-containing protein encoded by LRATCC53608_1774 and its homologues in pig clade IV and in three sourdough strains (Additional file 4). Although a putative pseudogene in these strains, it may still be translated as a protein ranging in size from about 880–2400 aa and exported by a non-classical secretion pathway (with a SecretomeP NN-score >0.5; [59]). DUF1542 repeats are found in many Gram-positive cell surface adhesins, such as MabA, a modulator of adhesion and biofilm formation, from *Lactobacillus rhamnosus* GG [60]. Homologues of this protein were not found in the two pig clade V strains and were also absent in all human strains but functional counterparts with a typical secretion signal were present in all rodent strains and one sourdough strain (Additional file 4). The significance of this DUF1542 repeat-containing surface protein in *L. reuteri* is as yet unknown but it appears to be conserved phylogenetically as either putative pseudogenes or functional genes in different host-derived strains.

#### The accessory SecA2-SecY2 secretion system

**Fig. 6:**
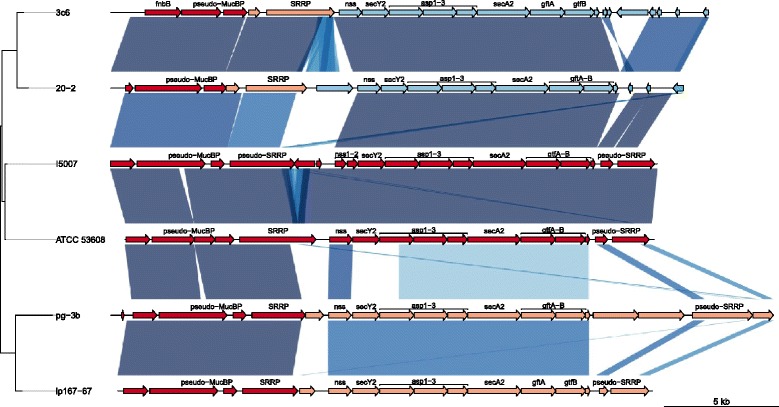
Phylogeny and BLAST comparisons of SecA2-SecY2 regions from the six pig strains of *L. reuteri*. For the four draft genomes of lp167-67, pg-3b, 20-2 and 3c6, the differently coloured gene bars represent separate contigs that were brought together after alignment to the completed reference ATCC 53608 genome using MUMmer. Genome comparisons are shown as BLAST matches, with the darker bars being of higher match. The phylogenetic tree was generated from the sequences only of the SecA2-SecY2 regions

In streptococci and staphylococci, the accessory SecA2-SecY2 system facilitates the selective export of glycosylated serine-rich repeat proteins (SRRPs) that often function as adhesins such as GspB, Fap1 and SraP [61–63]. This auxiliary protein secretion system is present in a limited number of Gram-positive bacteria and mycobacteria and other members of the Class Bacilli [64]; its sparse distribution among different species of lactobacilli implies that this system was horizontally acquired by only a few *Lactobacillus* lineages. In the *L. reuteri* rodent strain 100-23, an SRRP (Lr_70902) was identified as the only protein secreted by SecA2-SecY2 and shown to be essential for the formation of biofilm in germ-free mice [12]. The gene content within the accessory Sec cluster is conserved in *L. reuteri* ATCC 53608 when compared with that of rat strain 100-23 (Additional file 1: Figure S7). Furthermore, the SecA2-SecY2 cluster is also conserved in the other five pig strains (Fig. 6). It is of note that on the phylogenetic tree of Fig. 6, the SecA2-SecY2 clusters of ATCC 53608 and of I5007 fell between those of clade V strains 20-2 and 3c6 and the other clade IV strains pg-3b and lp167-67, with which ATCC 53608 and I5007 share the most backbone genetic material. This may indicate that the SecA2-SecY2 region of ATCC 53608 has undergone HGT from clade IV or that some amount of introgression is underway. HGT of the SecA2-SecY2 cluster in *L. reuteri* pig strains is supported by the presence of mobile genetic elements within the cluster of ATCC 53608 (LRATCC53608_0918-0921), a low GC content and by results from the analysis with Alien_hunter [65, 66] confirming that this region is likely to have been acquired by HGT, although it cannot be excluded that the presence of mobile elements is a prelude to deletion or modification of the cluster. By analogy to the 100-23 SRRP and to SRRPs from other bacteria, the SRRP homologue in ATCC 53608 (LRATCC53608_0906) and the other pig strains is likely to be secreted through this accessory pathway and indeed, this protein is found in extracellular extracts of ATCC 53608 (D. Latousakis and D. Kavanaugh, IFR, Norwich, UK, *personal communication*). LRATCC53608_0906, like Lr_70902 from 100-23, is unusually serine rich (25.2 and 36.1 % serine, respectively) and contains 11 of the predominant 10-amino acid repeat SLSNSVSMSE, compared with 91 such repeats in Lr_70902. However, each SRRP from the pig and rodent strains differs with respect to the number and sequences of their serine-rich repeats (Additional file 5). Comparing these repeat sequences, the SRRPs from clade IV strains ATCC 53608, lp167-67 and I5007 are more similar, while that from the other clade IV strain, pg-3b, is more like the SRRP from clade V strain 20-2. The serine residues of the SRRPs are likely to be glycosylated by glycosyltransferases associated with the SecA2-SecY2 cluster (LRATCC53608_0913 and LRATCC53608_0914).

No other functional large surface proteins are linked to the SecA2-SecY2 cluster in these pig strains, except for two LPXTG-containing pseudogenes that flank the cluster at either end (e.g. in strain ATCC 53608, LRATCC53608_0902-_0904 and LRATCC53608_0916-_0917). LRATCC53608_0902-_0904 is a MucBP-containing LPXTG pseudogene whereas LRATCC53608_0916-_0917 is an SRRP pseudogene that possibly arose from gene duplication of the functional SRRP, LRATCC53608_0906. In the completed genome of strain I5007 the SRRP itself is a pseudogene (LRI_0846-_0848) but not annotated as such (Fig. 6). Interestingly, this strain possesses a homologue (LRI_1680) of the CmbA (Lar_0958) mucus-binding protein that is found in many human isolates of *L. reuteri* [16, 17] but is absent in other pig strains. Also of interest, the MucBP-LPXTG pseudogenes from the pig strains have intact homologues in rodent and sourdough isolates of *L. reuteri*, suggesting their loss of function during evolution in the pig host (Additional file 6). The SRRPs showed high sequence conservation among pig strains, particularly in the N-terminal putative binding region (87–96 % aa identity; Additional file 5). Homology between the N-terminal domains of SRRPs from pig and rodent strains was lower (~50 % aa identity), suggesting that they may share a common structure but bind to different target ligands in their respective hosts. As expected, the pseudo-SRRPs showed more sequence diversity and were only about 30–40 % identical at the amino acid level to their respective functional counterparts (Additional file 7). Conservation of the SRRPs and SecA2-SecY2 cluster in all six *L. reuteri* strains from pig MLSA lineages IV and V (this work) and in most strains from rodents (MLSA lineages I and III) but not in isolates from human and poultry hosts (MLSA lineages II and VI) may explain host-specific differences in *L. reuteri* biofilm formation [12].

#### The EPS cluster

**Fig. 7:**
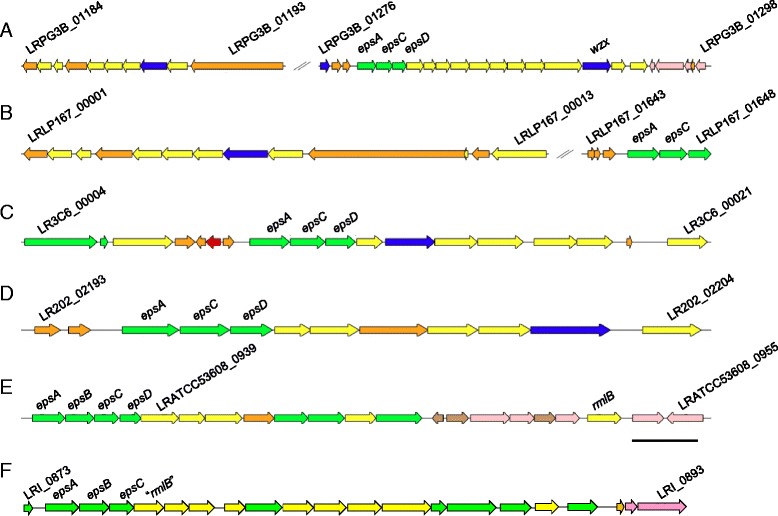
Predicted EPS gene clusters in the six *L. reuteri* pig strains. **a**, pg-3b, **b**, lp167-67, **c**, 3c6, **d**, 20-2, **e**, ATCC 53608 and **f**, I5007. Colour scheme: yellow, Gtf; green, other protein involved in polysaccharide biosynthesis; blue, membrane protein; orange, hypothetical protein; pink, transposase/mobile element protein; brown, pseudogene; red, RNA polymerase σ subunit

*L. reuteri* strains can produce EPS in several forms depending on the strain - levan (a β-2,6-linked fructan polymer) catalysed by the enzyme levansucrase (Lev, also known as fructosyltransferase, Ftf), inulin (β-2,1-linked fructooligosaccharides, FOSs, of varying chain length) catalysed by the enzyme inulosucrase (Inu) [13, 67, 68] and reuteran (a complex, branched 1–4, 1-6-α-D-glucan polymer) catalysed by glucosyltransferases (Gtf’s) GtfA or GtfO [69–71]. Ftf and Inu share 86 % aa similarity and are therefore difficult to distinguish at the sequence level. Some *L. reuteri* strains possess both types of enzyme but others have only one. Mutation of the single *ftf* gene in rodent strain 100-23 resulted in loss of EPS production and although the *ftf* mutant was able to colonise the murine GI tract of *Lactobacillus*-free mice in the absence of competition, colonisation was impaired in competition with the wild type, indicating a role in host interaction [72]. Genomic analyses of *L. reuteri* ATCC 53608 and the other five pig strains identified Ftf/Inu homologues in all six strains with approximately 80 % similarity at the protein level to that from rat strain 100-23 (LRATCC53608_1011, LRI_0973, LRLP167_01956, LR202_00872, LR3C6_01749 and LRPG3B_01561), suggesting that the *ftf*/*inu* gene was acquired by HGT early in the evolution of the species and subsequently diversified at accelerated rates. Homologues of Ftf/Inu are also present in human isolates of *L. reuteri*, ATCC 55730 [SD2112] and CF48-3a1. Recently, a cluster of more than 25 genes, including several encoding Gtf’s, that may contribute to EPS synthesis and is similarly organised compared to EPS-related genes described in other *Lactobacillus* strains, including *L. rhamnosus* GG [73], was reported in the *L. reuteri* human strain ATCC 55730 [SD2112] [74]. Although the start of this operon was similar in the human strain ATCC PTA-6475 [MM4-1a], the presence of an insertional element in strain ATCC PTA-6475 [MM4-1a] after the first 12 genes indicated that genomic rearrangements and gene loss had occurred [74]. Regions relating to these putative EPS gene clusters were found in the accessory genomes of the six pig strains (Fig. 7). Despite the close phylogenetic relationships between strains of the ATCC 53608 clade, the predicted EPS gene clusters showed poor conservation, with only the EpsA, C and D encoding genes strictly conserved, suggesting large-scale EPS gene cluster differences at the isolate level between these strains. This is in accordance with the EPS cluster in rodent *L. reuteri* strains, where the two EPS gene clusters show high variability [15]. Like the rodent strains, all six pig strains also possessed a second cluster of genes unlinked to the putative EPS cluster in Fig. 7—LRATCC53608_0648-_0660, LRI_0601-_0628, LR3C6_00497-_00506, LR202_01632-_01617, LRLP167_00566-_00570 and LRPG3B_01609-_01605 – four of which included a second EpsC-encoding gene and were therefore capable of producing other extracellular polysaccharides.

The glucansucrase/reuteransucrase GtfA responsible for reuteran biosynthesis was originally described in probiotic strain 121 [69] and an NCBI BLASTp analysis revealed the presence of homologues in sourdough strains TMW1.106 and TMW1.656 with 93 % aa identity to GtfA. The homologous GtfO [71] from human probiotic strains CF48-3a1 and ATCC 55730 [SD2112] are 68 % identical to GtfA. Other *L. reuteri* homologues Gtf180 and GtfML1 with 78–79 % aa identity to GtfA have been described in probiotic strain 180 and mouse strain ML1, synthesising α-1,6-linked dextran and α-1,3-linked mutan, respectively [71]. Interestingly, no homologues with significant (>40 %) aa identity to the above-mentioned four Gtf’s over the entire protein length were found in pig isolates ATCC 53608, 3c6 and 20-2. However, homologues from the other pig strains I5007, pg-3b and lp167-67 (LRI_0915, LRPG3B_00275 and LRLP167_01392, respectively) had 95–99 % aa identity to Gtf180, suggesting that these three clade IV strains most likely synthesise an α-1,6-linked dextran-type polymer. Like the *ftf* and *inu* genes, the *gtf180* homologues are unlinked to the putative EPS clusters displayed in Fig. 7, indicating that the latter are involved in the biosynthesis of other types of EPS. Thus, the ability of different strains to produce distinct types of EPS in the extracellular matrix in the host’s GIT may be a contributing factor in determining host specificity.

## Conclusions

This comparative genomics study provides novel insights into the ecology and evolution of the species *L. reuteri* with the porcine host. Together with previous phylogenetic analyses based on a small set of housekeeping genes, the genomic analysis highlights two divergent clades within *L. reuteri* pig strains that can be differentiated from other *L. reuteri* lineages. Although no genes were identified that were conserved among all pig isolates and specific to this host, we identified genes specific for and conserved among strains from the two pig phylogenetic lineages IV and V, with high rates of homology among strains. The two populations of porcine *L. reuteri* appear to have evolved separately, through a process driven by distinct selective pressures, e.g. different pig host genotypes or environmental factors such as dietary components, which resulted in the absence of common pig-specific genes among these two populations. However, from the phylogenetic analysis, the closeness of rodent clade III with pig clade IV strains, and of human clade VI with pig clade V strains could reflect an evolution of ancestral strains following contact of rodents and humans, respectively, with pig populations. The identification of several surface proteins, some with mucus-binding MucBP domains or other repeated domains found associated with adhesins, as being pig clade-specific might contribute to host-specificity, and ultimately to the clustering of strains away from other *L. reuteri* lineages. Clearly, these genes provide a basis for future functional studies on the ecology of *L. reuteri* in the pig GIT.

## Methods

### Strains

The origins of the *L. reuteri* strains used in this study are listed in Table 1.

### Genome sequencing, assembly and annotation

#### *Complete genome sequence of the pig* L. reuteri *strain ATCC 53608*

For the initial draft assembly [26] *L. reuteri* DNA had been used to generate in excess of 365 Mbp of sequence from a combination of shotgun and 3-kbp paired-end libraries (220 Mbp and 145 Mbp, respectively) on the 454 GS FLX sequencer (Roche) using the Titanium Chemistry. Reads passing the default filter settings had been assembled using gsAssembly V2.3 software (Roche) and had generated 13 scaffolds containing 99 large contigs (>500 bp) and spanning 1.96 Mbp of sequence. Standard PCR followed by primer walk sequencing on the resulting products was used to close the gaps located in scaffolds. Multiplex PCR was employed to identify adjoining contigs and respective primer pairs for which no linkage had been established previously and upon re-amplification under standard conditions the resulting products were analysed by primer walk sequencing. The sequence assembly was carried out using the Phred/Phrap [75] software in conjunction with the Staden package [76] and the expected error rate is around 1/500000 bp.

#### Bioinformatics analyses of ATCC 53608 genome

The finished *L. reuteri* ATCC 53608 sequence was annotated using the GenDB 2.4 annotation tool [77]. Protein-coding ORF sequences (CDS) were determined using Prodigal [44]. tRNA genes were identified with tRNAscan-SE [78]. An automatic functional annotation was computed in GenDB (CeBiTec) based on different analyses, and in addition, results from an automated RAST (Rapid Annotation using Subsystem Technology) [79] annotation were imported into the GenDB environment. The combined information available was employed for the manual annotation of each predicted gene. Pseudogenes were identified through a comparison of predicted gene products with respective proteins in the non-redundant peptide sequence database. Similarity searches [80] were performed against different databases, including the non-redundant and the Refseq protein [81] databases provided by National Centre for Biotechnology Information (NCBI), SWISS-PROT and TrEMBL [82], KEGG [83], Pfam [84] and TIGRFAM [85]. Additionally, SignalP [86, 87], PRED-LIPO [88, 89], LipoP [90–92], an additional lipoprotein pattern search [93] and TMHMM [94, 95] were applied to detect potential secretion signals, lipoproteins and transmembrane helical domains, respectively. Predicted ORF sequences were manually reviewed and alterations were made on the basis of the presence of potential ribosomal binding sites, sequence alignments and available literature data. Putative carbohydrate-active enzymes were identified with the methods used for the daily updates of the CAZy database [96, 97].

The two assigned plasmid sequences were confirmed as closed circular molecules by PCR with primer pairs B40/B41 (5’-CCGGTACGGTTTAAGTAGTC-3’ and 5’-TTGGAAAGTAACATCCATAGG-3’, respectively, for plasmid pI) and sc10-for/sc10-rev (5’-AACTGAAACCAATATACACTC-3’ and 5’-CTTAACAGAGTTATAGCCTCC-3’, respectively, for plasmid pII). The annealing temperatures used were 60 and 54 °C, respectively.

#### Sequencing of four draft genomes of L. reuteri strains isolated from pigs

DNA was extracted using the method of [14], purified further after treatment with DNase-free RNase to remove RNA and supplied to The Genome Analysis Centre (TGAC) for Illumina sequencing using the GAII platform. Paired-end sequencing was carried out using approximately 500–600 bp insert libraries. Between 45.6–50.7 × 10^6^ reads were collected for each sample, respectively, corresponding to a depth of coverage of approximately × 1800. Three alternative assemblies were created, set 1 was carried out using the Abyss assembler at TGAC, set 2 was created by Genostar, Montbonnot, France and set 3, from SequenceAnalysis.co.uk, Norwich, UK, corresponded to the second assembly with contaminating contigs of low coverage removed. Sequences from the Illumina marker phage, PhiX174, were detected in the draft assemblies and excluded from the final assembly. Annotation was built from a set of three inputs, a Genostar-based annotation platform, input from the RAST annotation program [79], Prokka rapid annotation [98] and with additional annotation from SequenceAnalysis.co.uk. Gene numbers were created for each strain with an included qualifier to link the gene numbering for conserved genes to that of *L. reuteri* ATCC 53608.

### Bioinformatics analyses of completed and draft *L. reuteri* genomes

#### Calculating the % average nucleotide identity (ANI)

The completed genome of strain ATCC 53608 was compared individually with each of the draft genomes of strains lp167-67, pg-3b, 3c6 and 20-2. ANIs were calculated using JSpecies [99].

#### Determining the two sets of pig clade-specific genes

Genes specific to the porcine phylogenetic lineages IV and V were identified using the Phylogenetic Profiler tool (which is based on pre-computed similarities between genes) implemented in the Integrated Microbial Genomes (IMG/ER) system of the Joint Genome Institute [46]. All 20 available completed and draft genomes of *L. reuteri* were included in the analysis, and genes were identified that had homologues in all strains from lineage IV or V, respectively, but not in strains from all other lineages, using a minimum percent identify of 50 % and a maximum E-value of 1e-5 (with the exclusion of pseudogenes).

In addition to the above analysis, a full reciprocal FASTA analysis was carried out and examined in the genome browser Artemis [47, 48]. A cut-off of 30 % identity over 80 % of the protein length was used. As a third alternative analysis to the above, all of the orthologous gene clusters were computed with OrthoMCL [39] default parameters and examined separately using the MySQL [100] database method and the GET_HOMOLOGUES programme [101].

#### Generation of maximum likelihood phylogenetic tree of L. reuteri strains based on full genome sequences

Twenty genome sequences of *L. reuteri* were aligned by Mugsy [102]. All the homologous blocks present in all genomes were extracted and concatenated using an in-house Perl script. All the disordered regions were trimmed by the TrimAl tool [103]. A maximum likelihood phylogenetic tree of the core genome alignment was built based on the best model (GTR + I + G) predicted by jModelTest [104] using PhyML software [105] with bootstrapping for 1000 replicates.

### Ethics statement

No animal ethics approval was required for this study.

### Availability of data and materials

All the sequence assembly data sets supporting the results of this article have been deposited at EMBL. The completed genome of *L. reuteri* ATCC 53608 has been assigned accession numbers LN906634, LN906635 and LN906636 for chromosome and plasmids pI and pII, respectively. Plasmid pLUL631 from *L. reuteri* 1063 has accession number HF570055. The annotated scaffolds of the four draft genomes have the following accession numbers: *L. reuteri* pg-3b (LN887201-LN887304), *L. reuteri* 3c6 (LN887305-LN887505), *L. reuteri* 20-2 (LN887506-LN887693) and *L. reuteri* lp167-67 (LN887694-LN887827).

## Additional files

Additional file 1:
**Figure S1A and B.**Plasmid profiles from strains of *L. reuteri*. **Figure S2.** IS element target site sequence logos from strain ATCC 53608. **Figure S3A and B.** *L. reuteri* pan-genomes displayed as frequency of orthologous gene clusters per number of genomes. **Figure S4.** Core and unique genes from 20 genome-sequenced strains of *L. reuteri*. **Figure S5.** Circular comparison of selected 13 genome-sequenced strains of *L. reuteri*. **Figure S6.** Conservation of putative surface proteins from ATCC 53608 with four other pig-derived strains. **Figure S7.** Comparison of accessory SecA2-SecY2 clusters from *L. reuteri* strains ATCC 53608 and 100-23. (PDF 1398 kb) Additional file 2: Table S1. IS elements in *L. reuteri* ATCC 53608. (XLSX 10 kb) Additional file 3: Table S2. Predicted surface proteins from *L. reuteri* ATCC 53608. (XLSX 14 kb) Additional file 4: Table S3. Homologues of DUF1542 repeat-containing cell wall protein LRATCC53608_1774 in genome-sequenced strains of *L. reuteri*. (XLSX 12 kb) Additional file 5: Table S4. Composition of 10-aa repeats in pig strain SRRPs compared with the rat 100-23 SRRP. (XLSX 11 kb) Additional file 6: Table S5.
*L. reuteri* homologues of SecA2-SecY2 cluster-associated MucBP-containing pseudogene protein LRATCC53608_0902-_0904. (XLSX 11 kb) Additional file 7: Table S6. Amino acid identity between pseudo-SRRPs from *L. reuteri* ATCC 53608 and 100-23 and functional SRRP counterparts. (XLSX 8 kb)

## Abbreviations

aa: amino acid
AFLP: amplified fragment length polymorphism
ANI: average nucleotide identity
CDS: protein-coding ORF sequence
CGH: comparative genomic hybridisation
CmbA: cell and mucus-binding protein
CRISPR: clustered regularly interspaced short palindromic repeat
EPS: exopolysaccharide
GI: gastrointestinal
HGT: horizontal gene transfer
MLSA: multi-locus sequence analysis
MLST: multi-locus sequence type
MUB: mucus-binding protein
ORF: open reading frame
PCR: polymerase chain reaction
RAST: rapid annotation using subsystem technology
SRRP: serine-rich repeat protein
TGAC: The Genome Analysis Centre
