# Supplementary material for: The pan-genome of Lactobacillus reuteri strains originating from the pig gastrointestinal tract
Source: BMC Genomics. 2015 Dec 1;16:1023. doi: 10.1186/s12864-015-2216-7 (PMC4667477; doi:10.1186/s12864-015-2216-7)
Supplement: Additional file 1: — Figure S1A and B.Plasmid profiles from strains of L. reuteri. Figure S2. IS element target site sequence logos from strain ATCC 53608. Figure S3A and B. L. reuteri pan-genomes displayed as frequency of orthologous gene clusters per number of genomes. Figure S4. Core and unique genes from 20 genome-sequenced strains of L. reuteri. Figure S5. Circular comparison of selected 13 genome-sequenced strains of L. reuteri. Figure S6. Conservation of putative surface proteins from ATCC 53608 with four other pig-derived strains. Figure S7. Comparison of accessory SecA2-SecY2 clusters from L. reuteri strains ATCC 53608 and 100-23. (PDF 1398 kb) [file 12864_2015_2216_MOESM1_ESM.pdf]

A.

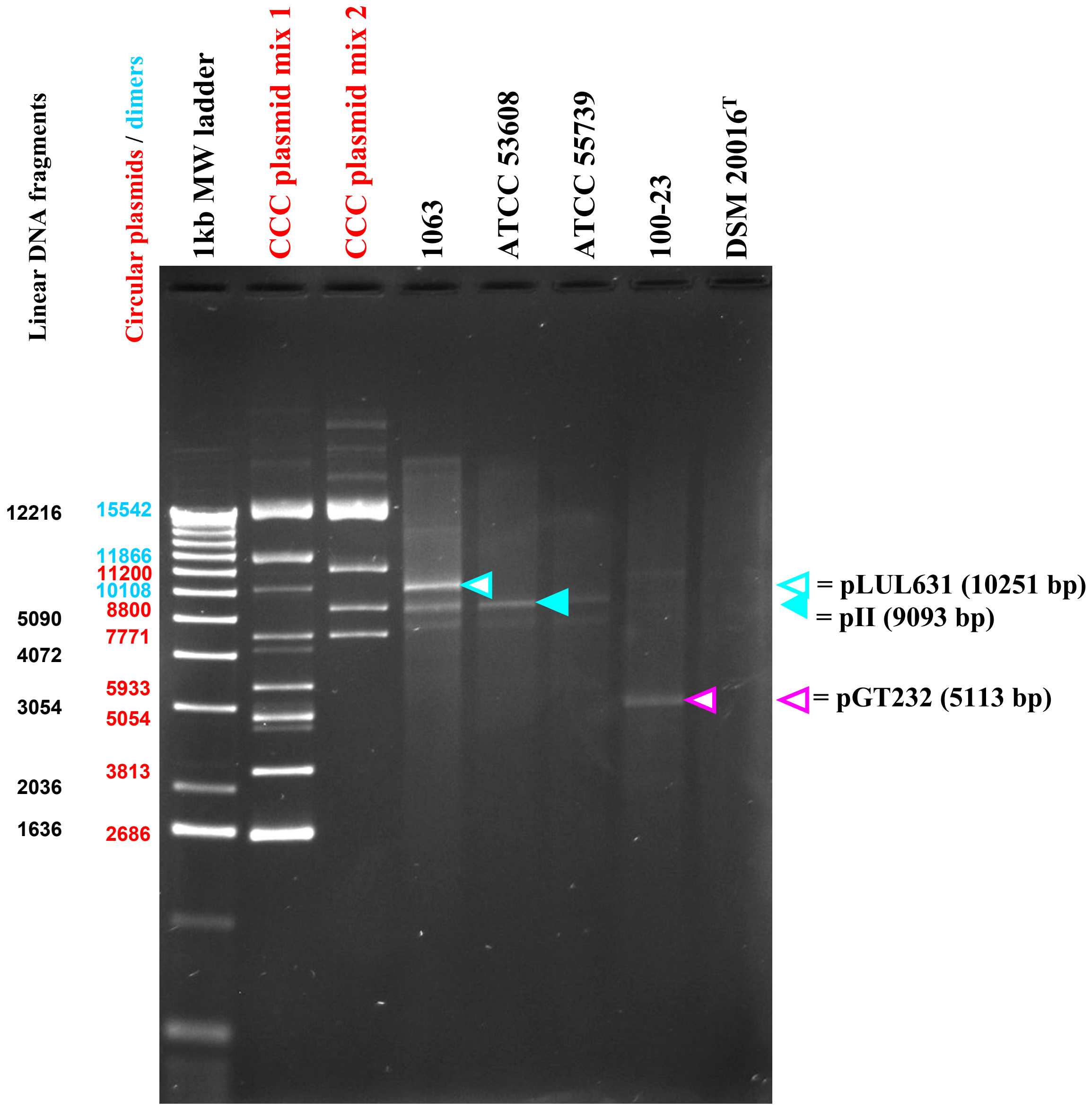

Supplementary Fig. S1A. Plasmid profiles from strains of *L. reuteri*. **A.** Plasmid DNAs isolated from related pig strains 1063 (parent of ATCC 53608), ATCC 53608 and ATCC 55739 (variant of ATCC 53608), rat strain 100-23 and human strain DSM 20016^T (a plasmid-minus strain). Sizes of covalently closed circular (CCC) plasmids were estimated from the mobility of mixtures of CCC plasmids of known size and related to that for linear DNA MW marker fragments to estimate CCC plasmid sizes in B.

B.

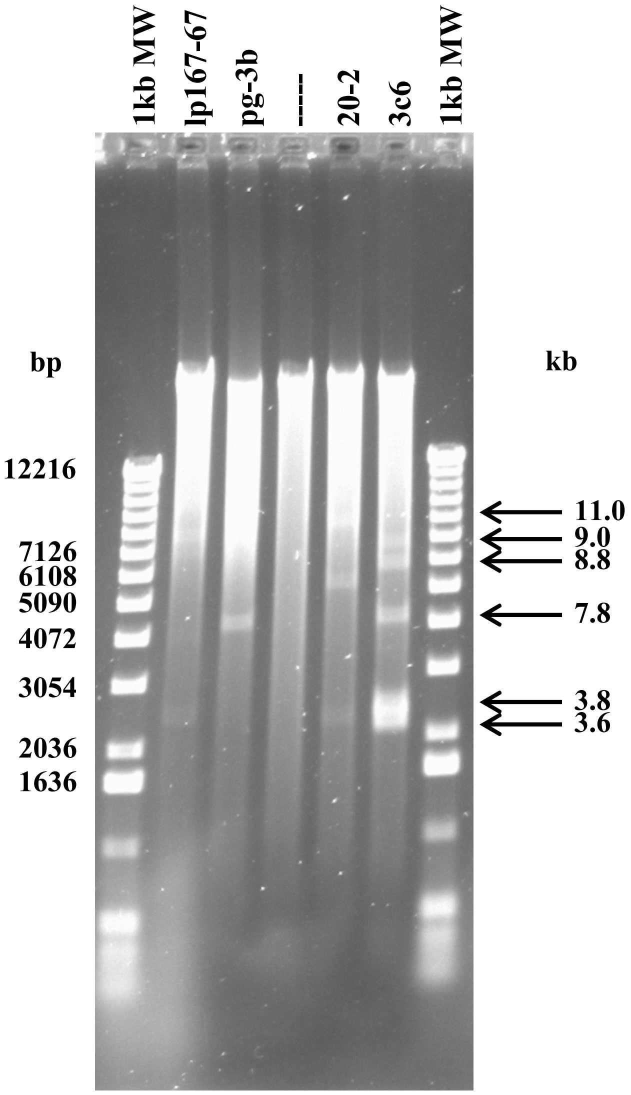

	Estimated circular plasmid size (kb)					
L. reuteri **strain**	**3.6**	**3.8**	**7.8**	**8.8**	**9.0**	**11.0**
lp167-67	+	+	—	—	—	—
pg-3b	—	—	+	—	—	—
20-2	+	+	—	+	—	—
3c6	+	+	+	—	+	+

Supplementary Fig. S1B. Plasmid profiles from strains of *L. reuteri*. **B.** Total genomic and plasmid DNA was isolated from pig-derived strains as described in Methods. Putative plasmids indicated by labelled arrows. - - - -, DNA isolated from a non-*L. reuteri* strain.

LRATCC53608_0045

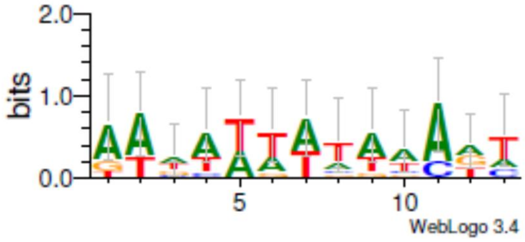

LRATCC53608_0105

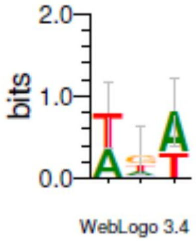

LRATCC53608_0137

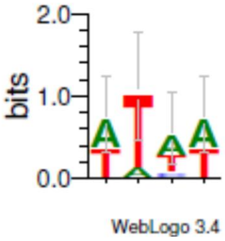

LRATCC53608_0176

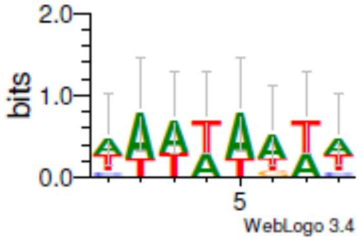

LRATCC53608_0425

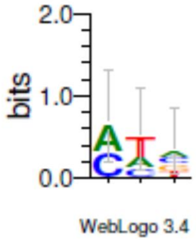

Supplementary Fig. S2. IS element target site sequence logos from strain ATCC 53608.

A. **B.**

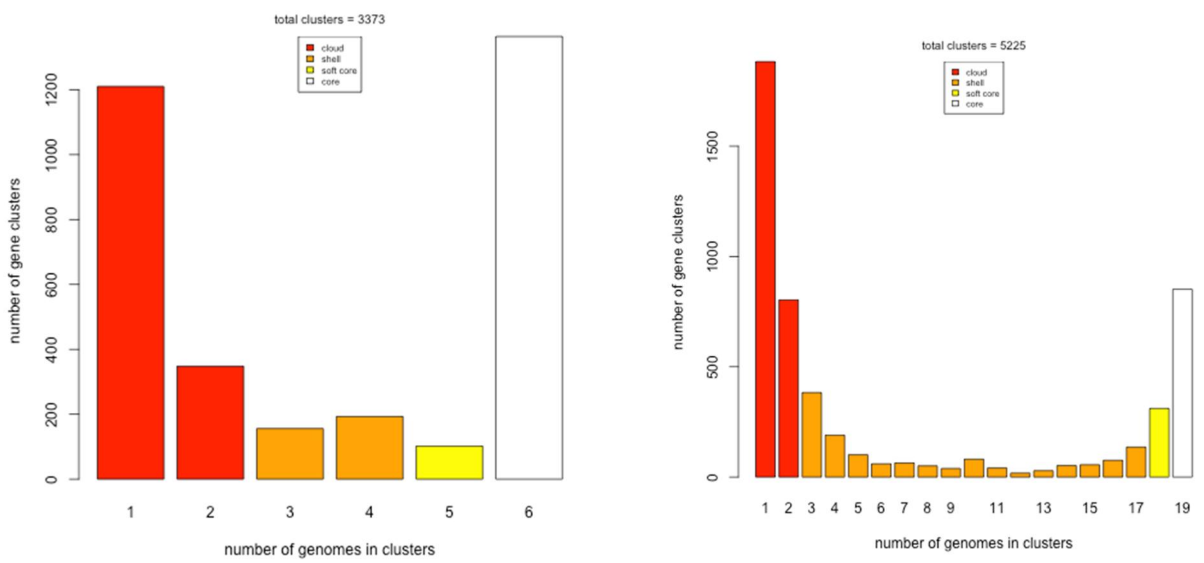

Supplementary Fig. S3A and B. *L. reuteri* pan-genomes displayed as frequency of orthologous gene clusters per number of genomes. **A.** *L. reuteri* pan-genome of the six pig strains, **B.** *L. reuteri* pan-genome of 19 strains from different hosts, including the six from pig. In each case, the core represents orthologous gene clusters present in all of the genomes considered in the analyses, the soft core represents gene clusters included in 95% of all the considered genomes (*viz.* 5/6 genomes in **A** and 18/19 genomes in **B**), the cloud represents gene clusters present only in one or two of the considered genomes whilst the shell represents the remaining gene clusters present in several of the genomes.

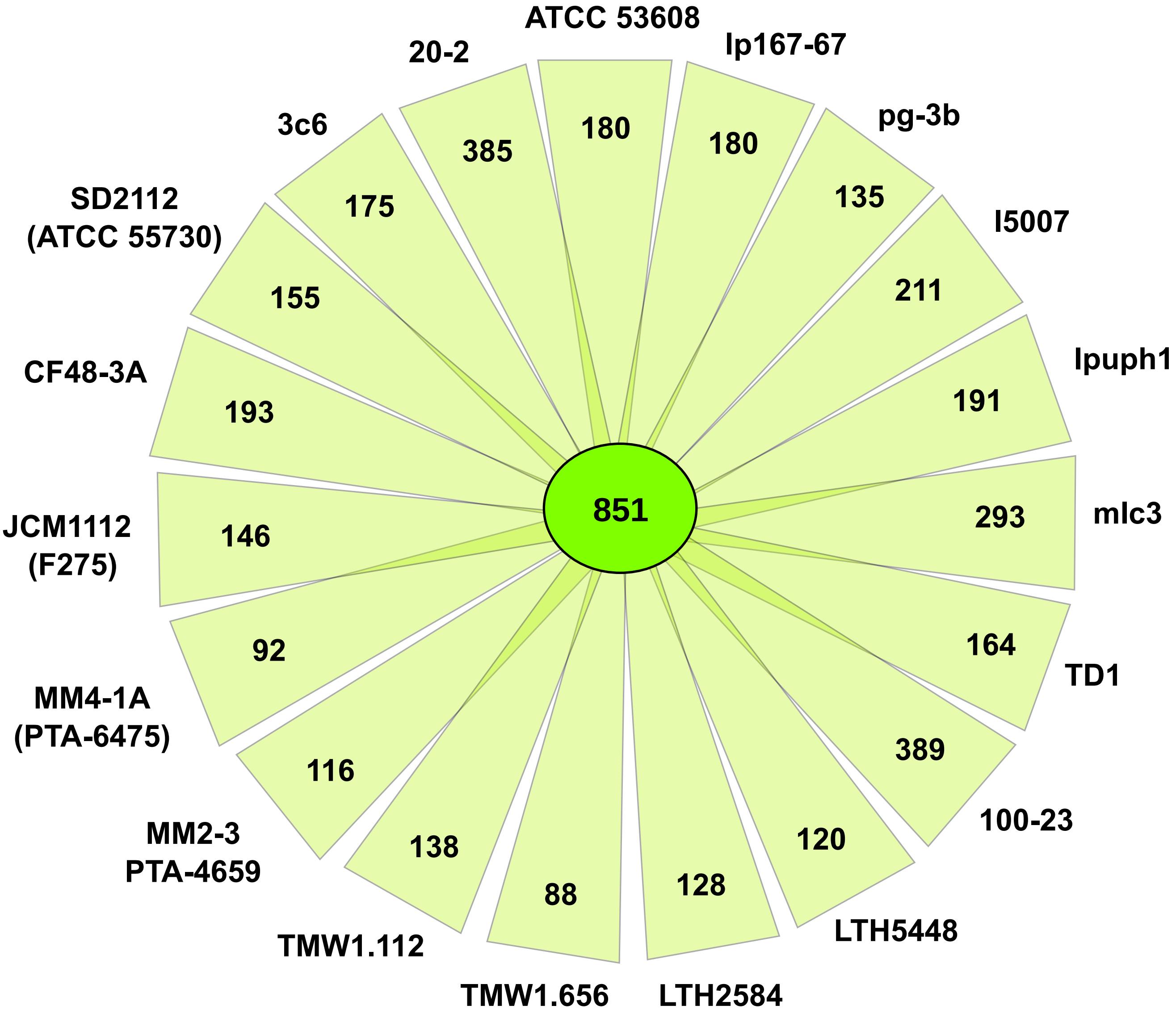

Supplementary Fig. S4. Core and unique genes from 19 genome-sequenced strains of *L. reuteri.* The number of core genes, expressed as the number of orthologous gene clusters per genome, is indicated at the centre and of the unique genes (3479 in total) indicated towards the outside of the figure for each genome. JCM1112 and DSM20016 were considered as a single genome, indicated as JCM1112 (F275).

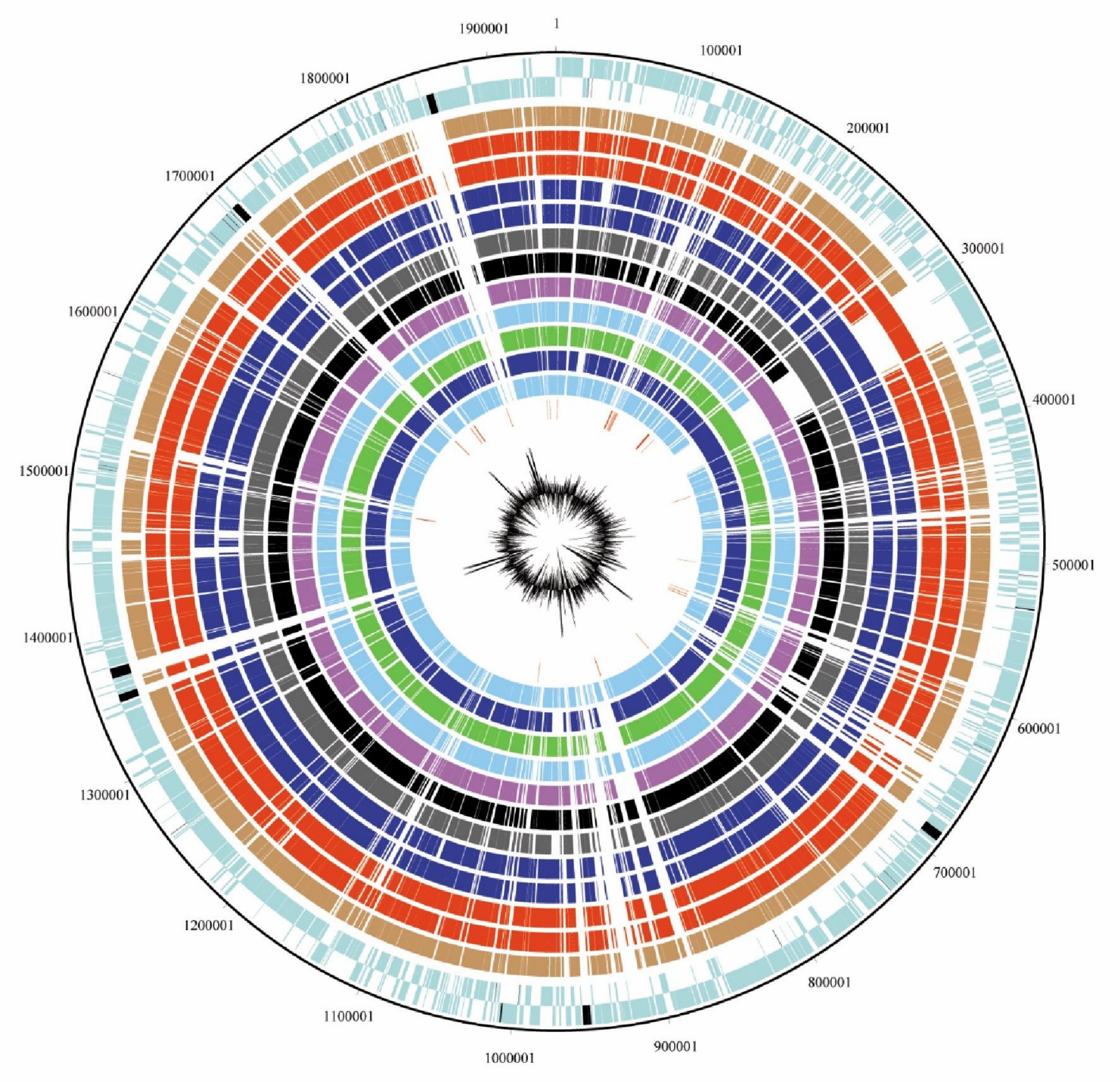

<u>Outermost to innermost circles</u>:

1. ATCC 53608 forward strand (light blue)
2. ATCC 53608 reverse strand (light blue)
3. I5007 (brown)
4. lp167-67 (red)
5. pg-3b (red)
6. 3c6 (royal blue)
7. 20-2 (royal blue)
8. mlc3 (grey)
9. 100-23 (black)
10. ATCC PTA-6475 [MM4-1A] (purple)
11. lpuph1 (pale blue)
12. ATCC PTA-4659 [MM2-3] (green)
13. ATCC 55730 [SD2112] (blue)
14. TD1 (pale blue)
15. Clade-specific CDSs (pink)
16. GC content (black)

Supplementary Fig. S5. Circular comparison of selected 13 genome-sequenced strains of *L. reuteri***.**

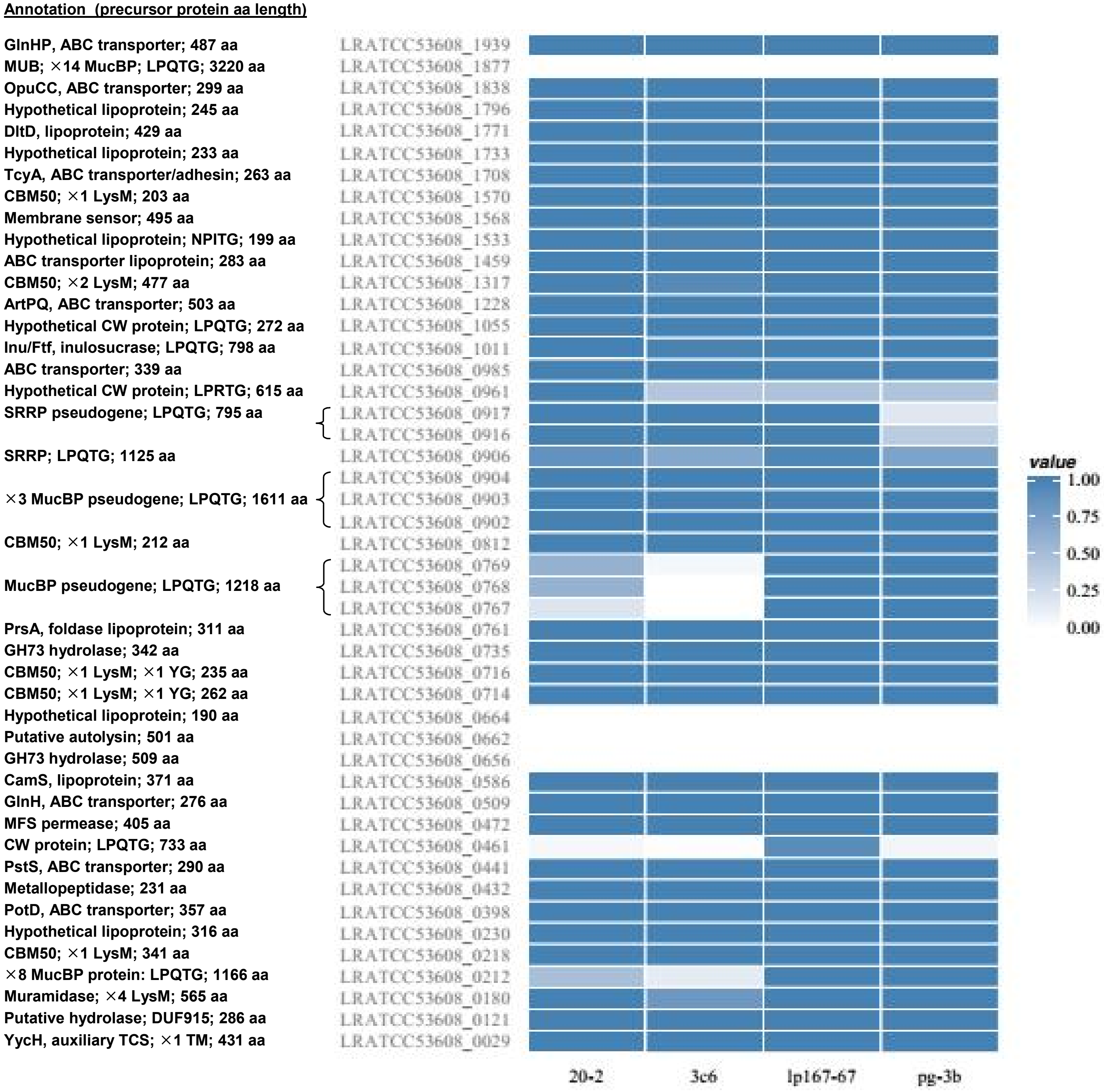

Supplementary Fig. S6. Conservation of putative surface proteins from ATCC 53608 with four other pig-derived strains. The heatmap is based on DNA sequence read alignments with the ATCC 53608 genome across draft genomes 20-2, 3c6, lp167-67 and pg-3b. A value of 1.0 corresponds to 100% gene coverage of the DNA sequence of the predicted surface protein's DNA sequence in ATCC 53608 by alignments of Illumina sequence reads present in the draft genome sequences of the four strains. The darker the colour, the higher the degree of coverage of the genes.

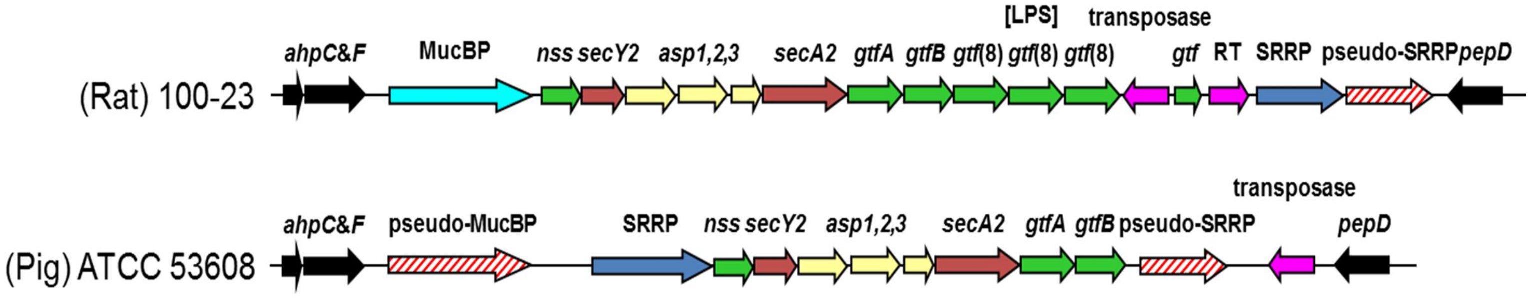

Supplementary Fig. S7. Comparison of accessory SecA2-SecY2 clusters from *L. reuteri* strains ATCC 53608 and 100-23.
